# Evaluation of antibiotics resistance in Southern Iran in light of COVID‐19 pandemic: A retrospective observational study

**DOI:** 10.1002/hsr2.1153

**Published:** 2023-03-15

**Authors:** Rahim Raoofi, Negin Namavari, Vahid Rahmanian, Mohammad Hadi Dousthaghi

**Affiliations:** ^1^ School of Medicine, Department of Infectious Diseases Jahrom University of Medical Sciences Jahrom Iran; ^2^ School of Medicine Jahrom University of Medical Science Jahrom Iran; ^3^ Department of Public Health Torbat Jam Faculty of Medical Sciences Torbat Jam Iran; ^4^ Biosystems Engineering Faculty Tarbiat Modares University Tehran Iran

**Keywords:** antibiotic resistance, COVID‐19, Gram‐negative bacteria, nosocomial infection, outbreak

## Abstract

**Background and Aims:**

Antimicrobial resistance (AMR) was taken as one of the high‐priority long‐lasting public health issues, although it might have been underrated in terms of COVID‐19 pandemic emergence. Regarding limited data on assessing the pandemic effect on AMR trend in Iran, this study aimed to describe the epidemiology of antibiotics resistance during the COVID pandemic in southern Iran.

**Methods:**

This descriptive study was conducted on 2675 patients' samples collected and processed in a referral COVID‐19 center hospital in southern Iran from March 21, 2019, to February 18, 2020 (prepandemic), and February 19, 2020, to March 21, 2021 (pandemic). Susceptibility test results in sensitivity and resistance levels were compared in prepandemic and pandemic periods.

**Results:**

Compared to prepandemic, the inpatient number has increased almost three times. On the other hand, there are around four times fewer outpatients now. More than 85% of the specimens were found in urine samples. In all, 92.22% of all bacteria samples were Gram‐negative isolates, with *Escherichia coli* accounting for 59.19% of them. The change rate of Gram‐negative bacteria resistance to antimicrobials is an average of 7.74% (*p* < 0.001). On the other hand, the average change rate of Gram‐positive bacteria resistant to antibiotics has decreased by 19.3% (*p* = 008). As a forerunner among other Gram‐negative bacteria, the average change rate for *Pseudomonas aeruginosa* and *Klebsiella pneumonia* resistance to monitored antibiotics was 89% and 66.3%, respectively (*p* < 0.001).

**Conclusion:**

During the Covid‐19 pandemic, the increase in AMR among Gram‐negative bacteria, particularly *P. aeruginosa* and *K. pneumonia*, was observed compared to the prepandemic. This further limits treatment options, and endangers global public health.

## 1 INTRODUCTION

Antimicrobial resistance (AMR), as one of the most critical worldwide public health issues, should be intercepted as soon as possible.^1^ AMR affects health care, and life quality eventuating in death and extra cost.^2^ If there are no interventions, it was estimated that the annual death rate will reach 10 million in 2050 caused by AMR.^3^ Considering the pathogen's resistance does not have any geographical boundary, AMR must not be taken as a bordered problem for just some countries or regions regarding either income or level of development.^4^

In 2017, World Health Organization listed some high priority, and critical bacteria most of which belonged to Gram‐negative bacteria. These pathogens have multidrug‐resistant features and cause healthcare‐associated infections.^5^

The COVID‐19 pandemic as a parallel issue to AMR is taken as a crucial health emergency. It is an acute problem; on the other hand, the AMR is the long‐lasting one.^6^ Some comparative studies on AMR rates during COVID‐19 and before the pandemic has disclosed a significant change.^7^, ^8^, ^9^, ^10^, ^11^

Taking action to slow down the spread of COVID‐19, such as social distancing, using physical barriers, and so forth, has led to a reduction in the spread of other infections, which resulted in less usage of antimicrobials. Hence, it was reported that patients with other infections prefer not to seek care in healthcare centers.^12^, ^13^, ^14^ On the other hand, researchers empirically reused some medications, including some antibiotics, regarding their antiviral effects, to treat COVID‐19 patients, disregarding antimicrobial stewardship rules.^5^, ^12^, ^13^, ^14^ For instance, azithromycin was prescribed to treat SARS‐CoV‐2. If their usage has not had any significant effect on treating COVID‐19, this matter has not had any consequence, but the AMR increased.^9^ The bacterial co‐infection with COVID‐19 has increased the rate of antibiotic prescription in hospitalized patients, but there are no data on community antibiotic usage.^15^, ^16^, ^17^

The mutual effect of AMR and COVID‐19 is unknown yet.^6^ This study aimed to describe the epidemiology of antibiotics resistance in Jahrom District, Southern Iran during the COVID pandemic.

## 2 METHOD

### 2.1 Study design

This is a descriptive (retrospective observational) study in which data were collected from either inpatients or outpatients at a COVID‐19 referral hospital affiliated with Jahrom university of medical sciences. A total of 2675 patient samples were processed from March 21, 2019, to February 18, 2020 (prepandemic), and February 19, 2020, to March 21, 2021 (pandemic) among all referred patients to the hospital. In Iran, the pandemic officially started in mid‐February 2020. The data were divided into two categories: prepandemic and pandemic periods.

The clinical specimens of any positive culture of urine, blood, sputum, stool, wound, cerebrospinal fluid, aspiration, and pleural fluid/bronchoalveolar lavage (BAL) which had recorded an antibiogram were included. Any culture which had not have a recorded antibiogram was excluded.

### 2.2 Sample identification and disc diffusion susceptibility testing method

Positive cultured samples in sterile saline were incubated to reach 0.5 McFarland (1.5 × 10^8^ colony‐forming unit/mL) concentration. The colonies were incubated in Mueller‐Hinton agar for 24–48 h, depending on the sample type, which had been at 35–37°C. According to Clinical and Laboratory Standards Institute (CLSI) 2020 18 guidelines, the disc diffusion technique was used to assess the sensitivity of bacteria isolated from patient samples at two sensitive and resistant levels by measuring the size of the antibiotic disc's inhibitory growth zone. To identify the strains standard biochemical tests were done.

The antibiotics discs (PadtanTeb) containing CN: cephalexin 30 µg, CP: ciprofloxacin 5 µg, CRO: ceftriaxone 30 µg, CTX: cefotaxime 30 µg, FEP: cefepime 30 µg, GM: gentamicin 10 µg, SXT: cotrimoxazole 1.25/23.75 µg, VA: vancomycin 30 µg.

### 2.3 Data collection

The hospital medical records have been the basis of clinical features. The data were entered into an electronic pattern. The considered independent factors of the studied population have been age, sex, antibiotics, either sensitivity or resistance to antibiotics, and hospitalization status.

### 2.4 Statistical analysis

IBM SPSS version 21 software was used for data analysis. The participants' demographic characteristics were expressed in frequency and percent. The association between antibiotic resistance and study variables was assessed using the chi‐square test and Student's *t*‐test at a significance level of 0.05. The change rate indicated by the difference level between prepandemic and the pandemic period of time divided by its frequency during the prepandemic period of time as what is shown by the following equation:

(1) $$\text{change rate}\;=\frac{\;N_{\mathrm{Pan}}-N_{\mathrm{pre}}}{N_{\mathrm{pre}}},$$

where *N*_pan_ is the number of frequency during the pandemic period of time and *N*_pre_ the number of frequency during prepandemic period of time.

Supposing that the bacteria behavior about the evaluated antibiotics is independent, a weighted average was reported as overall monitored bacteria resistance to all evaluated antibiotics by the following equation:

(2) $$\text{overall resistance}\;=\frac{\;\left(R_{1}\times n_{1}\right)+\left(R_{2}\times n_{2}\right)+\;\text{⋯}\;+\left(R_{n}\times n_{n}\right)}{n_{1}+n_{2}+\;\text{⋯}\;+n_{n}},$$

where *R*_1_ stands for the antibiotic resistance of supposed isolated bacteria to first antibiotic and *n*_1_ is the total sample number of the same isolated bacteria for which antibiogram test is reported for the first antibiotic. Moreover, *R*_2_ stands for the antibiotic resistance of the same isolated bacteria to the second antibiotic, and *n*_2_ is the total sample number of the same isolated bacteria for which the antibiogram test is reported for the second antibiotic. *R*_*n*_ stands for the antibiotic resistance of the same isolated bacteria to the *n*th antibiotic and *n*_*n*_ is the total sample number of the same isolated bacteria for which an antibiogram test is reported for the *n*th antibiotic. To establish an indicator to track changes in bacterial resistance patterns in light of the COVID‐19 presence, overall resistance has been calculated. A *p v*alue <0.05 was considered a significant level.

### 2.5 Ethics approval

This research project was approved by the Ethics Committee of the Jahrom University of Medical Science, Fars, Iran (IR.JUMS.REC.1398.093).

## 3 RESULT

### 3.1 Clinical specimens and isolated pathogens and demographic features

The study population included 2675 patient samples, of whom 1778 and 897 were for prepandemic and pandemic, respectively. As compared to prepandemic, during the pandemic the inpatients' mean age had no significant change (prepandemic versus during the pandemic, 60.2 ± 22.90 versus 58.86 ± 17.59, *p* = 0.124). Unlike inpatients, the number of outpatients decreased. Although both genders had an equal inpatient portion each year, female patients possess more than 70% of outpatients every year (Table 1).

**Table 1:** Demographic characteristics of patients in prepandemic and the pandemic periods.

	Admission type	Prepandemic^a^	Pandemic^b^	Change rate	*p* Value
Sample number	Inpatient	155	470	203.23%	<0.001^c^
	Outpatient	1623	427	−73.69%	
Mean age	Inpatient	60.2	58.86	−2.23%	0.124^d^
	Outpatient	45.72	46.93	2.65%	0.114^d^
Female	Inpatient	82	241	193.90%	0.164^c^
	Outpatient	1187	335	−71.78%	
Male	Inpatient	73	229	213.70%	0.124^c^
	Outpatient	436	92	−78.90%	

^a^ From March 21, 2019 to February 18, 2020.

^b^ February 19, 2020 to March 21, 2021.

^c^ Chi‐square.

^d^ Student's *t*‐test.

The urine samples contained the most specimens, followed by sputum, blood, wound, aspiration, pleural fluid and BAL, cerebrospinal fluid, and stool (Table 2).

**Table 2:** Demographic isolated from cultures in hospitals, Jahrom from 2019 to 2020.

Specimens	Prepandemic^a^		Pandemic b		Total	
Urine	1556	87.5%	724	80.71%	2280	85.23%
Sputum	76	4.3%	105	11.71%	181	6.77%
Blood	51	2.9%	41	4.6%	92	3.4%
Wound	62	3.5%	10	1.1%	72	2.7%
Aspiration	19	1.1%	5	0.6%	24	0.9%
Pleural fluid/BAL	8	0.4%	8	0.9%	16	0.6%
CSF	4	0.2%	2	0.2%	6	0.2%
Stool	2	0.1%	2	0.2%	4	0.1%
Total	1778	897	2675			

Abbreviations: BAL, bronchoalveolar lavage; CSF, cerebrospinal fluid.

^a^ From March 21, 2019, to February 18, 2020.

^b^ February 19, 2020 to March 21, 2021.

The most frequent Gram‐negative and Gram‐positive pathogens isolated in both years were *Escherichia coli* and *Staphylococcus aureus* specimens, respectively (Table 3). The order of the most prevalent bacteria was *E. coli*, *Pseudomonas aeruginosa*, *Staphylococcus* strains, *Klebsiella pneumonia*, *Citrobacter*, and *Acinetobacter baumannii* (Table 3).

**Table 3:** Pathogens isolated from cultures.

Bacteria	Prepandemic^a^		Pandemic^b^		Total	
*Escherichia coli*	547	30.76%	306	34.11%	853	31.89%
*Pseudomonas aeruginosa*	168	9.45%	114	12.71%	282	10.54%
*Staphylococcus* strains	57	3.2%	50	5.6%	107	4.00%
*Klebsiella pneumoniae*	46	2.6%	44	4.9%	90	3.4%
*Citrobacter freundii*	35	2%	10	1.1%	45	1.7%
*Proteus*	25	1.4%	13	1.4%	38	1.4%
*Acinetobacter baumannii*	13	0.7%	1	0.1%	14	0.5%
*Enterobacter*	4	0.2%	0	0%	4	0.1%
*Streptococcus*	3	0.2%	2	0.2%	5	0.2%
*Salmonella*	0	0%	2	0.2%	2	0.1%
*Shigella*	1	0.1%	0	0%	1	0.04%
Fungi	51	2.9%	47	5.2%	98	3.7%
Yeast	122	6.86%	50	5.6%	172	6.43%
Mix growth^c^	637	35.83%	258	28.76%	895	33.46%
No growth	69	3.9%	0	0%	69	2.6%
Total	1778	897	2675			

^a^ From March 21, 2019, to February 18, 2020.

^b^ February 19, 2020 to March 21, 2021.

^c^ Mix growth means that the culture showed a heavy growth of at least three organisms; this represents contamination of the urine with the patient's flora during collection.

Gram‐negative and ‐positive bacteria frequencies were 1329 and 112, respectively. *E. coli*, *P. aeruginosa*, and *K. pneumonia* were mainly detected from urine specimens. Moreover, *Staphylococcus* strains were predominant in blood and urine specimens. Furthermore, *Citrobacter* detection was more frequent in sputum specimens. *A. baumannii* was detected equally from sputum and blood specimens (Table 4).

**Table 4:** The most prevalent bacteria isolated across various clinical specimens' distribution.

Bacteria	Specimen								
	Urine	Sputum	Blood	Wound	Aspiration	Pleural fluid/BAL	CSF	Stool	Total
*Escherichia coli*	34.96% (797)	8.29% (15)	31.5% (29)	8.3% (6)	20.83% (5)	6.2% (1)	‐	‐	31.89% (853)
*Pseudomonas aeruginosa*	9.30% (212)	22.10% (40)	13% (12)	13.9% (10)	(0)	25% (4)	66.7% (4)	‐	10.54% (282)
*Staphylococcus strains*	1.36% (31)	3.87% (7)	32.6% (30)	31.9% (23)	37.5% (9)	31.2% (5)	33.3% (2)	‐	4.00% (107)
*Klebsiella pneumoniae*	3.51% (80)	3.31% (6)	1.1% (1)	1.4% (1)	(0)	12.5% (2)	‐	‐	3.36% (90)
*Citrobacter freundii*	0.04% (1)	8.29% (15)	10.9% (10)	18.1% (13)	16.7% (4)	6.2% (1)	‐	25% (1)	1.68% (45)
*Proteus*	1.62% (37)	(0)	1.1% (1)	‐	‐	‐	‐	‐	1.42% (38)
*Acinetobacter baumannii*	‐	3.87% (7)	7.6% (7)	‐	‐	‐	‐	‐	0.52% (14)
*Enterobacter*	‐	‐	2.2% (2)	2.8% (2)	‐	‐	‐	‐	0.15% (4)
*Streptococcus*	0.22% (5)	‐	‐	‐	‐	‐	‐	‐	0.19% (5)
*Salmonella*	‐	‐	‐	‐	‐	‐	‐	50% (2)	0.07% (2)
*Shigella*	‐	‐	‐	‐	‐	‐	‐	25% (1)	0.04% (1)
Fungi	0.13% (3)	39.23% (71)	‐	23.6% (17)	20.8% (5)	12.5% (2)	‐	‐	3.66% (98)
Yeast	6.67% (152)	9.94% (18)	‐	‐	4.2% (1)	6.2% (1)	‐	‐	6.43% (172)
Mix growth	39.17% (893)	1.10% (2)	‐	‐	‐	‐	‐	‐	33.46% (895)
No growth	3.03% (69)	‐	‐	‐	‐	‐	‐	‐	2.58% (69)
Total	2280	181	92	72	24	16	6	4	2675

Abbreviations: BAL, bronchoalveolar lavage; CSF, cerebrospinal fluid.

### 3.2 Resistance profile for the isolate bacteria and the effect of COVID‐19

A significant overall resistance increase among Gram‐negative bacteria was observed, *E. coli* excluded. *P. aeruginosa* and *K. pneumonia* had a prominent overall resistance increase, *Citrobacter* for that matter, but in a milder trend. Although *A. baumannii* resistance has not had a significant increase, its resistance level was 100%, after the pandemic. Superficially, it might be interpreted that *E. coli* resistance has overall decreased. However, better scrutiny discloses that its resistance to gentamicin, cefepime, and cefotaxime has increased (Table 5).

**Table 5:** Comparison of some Gram‐negative bacteria resistant to some antibiotics during prepandemic^a^ and pandemic^b^ periods of times.

Bacteria	COVID‐19 presence	CP% (*n*)	GM% (*n*)	CRO% (*n*)	SXT% (*n*)	FEP% (*n*)	CTX% (*n*)	Overall resistance
*Pseudomonas aeruginosa*	Prepandemic	37.88% (132)	28.57% (133)	NR	NR	69% (29)	NR	36.73%
	Pandemic	67.7% (65)	65.1% (63)	NR	NR	82.8% (29)	NR	69.43%
	*p* Value**	<0.001	<0.001	NR	NR	0.049	NR	<0.001
*Klebsiella pneumoniae*	Prepandemic	17.9% (28)	11.1% (27)	35.7% (28)	31.6% (19)	30% (10)	30% (10)	24.59%
	Pandemic	41.4% (29)	29% (31)	48.4% (31)	15.4% (13)	53.8% (13)	60% (15)	40.91%
	*p V*alue**	0.013	0.041	0.166	0.004	0.117	0.041	<0.001
*Acinetobacter baumannii*	Prepandemic	50% (10)	91.7% (12)	91.7% (12)	100% (3)	87.5% (8)	87.5% (8)	87.5%
	Pandemic	100% (1)	100% (1)	100% (1)	*	*	100% (1)	100%
	*p* Value**	0.006	0.03	0.03	*	*	0.07	0.149
*Citrobacter freundii*	Prepandemic	51.4% (35)	47.1% (34)	62.9% (35)	77.8% (9)	65.4% (26)	69.2% (26)	69.2%
	Pandemic	66.7% (9)	66.7% (9)	66.7% (9)	* (0)	83.3% (6)	75% (8)	75%
	*p* Value**	<0.001	<0.001	<0.001	*	<0.001	<0.001	<0.001
*Escherichia coli*	Prepandemic	41.71% (386)	25.26% (384)	51.41% (391)	54.15% (277)	59.62% (104)	58.88% (107)	44.51%
	Pandemic	40.76% (238)	29.07% (227)	51.26% (238)	39.06% (128)	63.4% (71)	61.40% (114)	44.29%
	*p* Value**	<0.001	<0.001	<0.001	<0.001	<0.001	0.403	<0.001
Gram negatives^c^	Prepandemic	40.44% (591)	27.97% (590)	52.01% (598)	52.42% (414)	61.58% (177)	61.45% (179)	45.15%
	Pandemic	46.79% (342)	36.85% (331)	59.03% (349)	40.66% (150)	68.07% (119)	68.55% (194)	51.58%
	*p* Value**	<0.001	<0.001	<0.001	<0.001	<0.001	0.017	<0.001

*Note*: Gram‐positive bacteria have a far smaller reported sample size than Gram‐negative bacteria. Although certain antibiotic use has increased dramatically in particular circumstances, the prevalence of Gram‐positive bacteria as a whole is significantly declining (Table 6).

Antibiotic not tested is indicated by *, ** chi‐square.

Abbreviations: CP, ciprofloxacin; CRO, ceftriaxone; CTX, cefotaxime; FEP, cefepime; GM, gentamycin; NA, not applicable according to CLSI 2020; SXT, cotrimoxazole.

^a^ From March 21, 2019 to February 18, 2020.

^b^ February 19, 2020 to March 21, 2021.

^c^ The cumulative status of all the above Gram‐negative microorganisms has been reported as their behavior.

**Table 6:** Comparison of some Gram‐positive bacteria resistant to some antibiotics during prepandemic^a^ and pandemic^b^ periods of times.

Bacteria	COVID‐19 status	SXT% (*n*)	CN% (*n*)	VA% (*n*)	Overall resistance
Gram positives^c^	Prepandemic	71% (7)	59% (22)	5.9% (17)	41.3% (46)
	Pandemic	100% (1)	64.3% (14)	5.6% (18)	33.3% (33)
	*p* Value**	0.099	0.026	0.808	0.008
*Staphylococcus coagulase‐negative*	Prepandemic	100.0% (1)	100.0% (7)	0.0% (7)	53.3% (15)
	Pandemic	100.0% (1)	83.3% (6)	10.0% (10)	41.2% (17)
	*p* Value**	‐	0.180	0.229	0.307
*Staphylococcus aureus*	Prepandemic	67% (6)	40% (15)	10% (10)	35% (31)
	Pandemic	*	50% (8)	0% (8)	25% (16)
	*p* Value**	*	0.014	0.438	<0.001

*Note*: Antibiotic not tested is indicated by *, ** chi‐square.

Abbreviations: CN, cephalexin; SXT, cotrimoxazole; VA, vancomycin.

^a^ From March 21, 2019 to February 18, 2020.

^b^ February 19, 2020 to March 21, 2021.

^c^ The cumulative status of all the above Gram‐positive microorganisms was reported as their behavior.

## 4 DISCUSSION

One of the future challenging public health issues as a subsequence of the COVID‐19 pandemic may have been an increase in AMR caused by indiscriminative antibiotic use. To the best of our knowledge, this is the first study evaluating the COVID‐19 pandemic's probable effect on AMR in Iran during the pandemic compared to prepandemic. Although there might be differences in the healthcare system set‐up of each country. It shows an overall increase in AMR by the pandemic presence, which has been shown in some other countries, including India,^8^ Mexico,^18^ Indonesia,^10^ Serbia,^11^ and so forth.

Considering the hospital was a COVID‐19 referral center, intensification in the inpatient admission, 203.69%, could be taken as a consequence of the COVID‐19 pandemic, but, by contrast, a drastic fall in the outpatients, 73.69%, shows the avoidance of referring to hospital during the pandemic, unless it were emergency.^7^, ^19^ Mandatory lock‐down to create social distancing, personal fear of the contagious pandemic in the healthcare centers, lack of knowledge, concluded in preferring over‐the‐counter usage of antibiotics instead of referring to healthcare centers; moreover, remote medicine, the lack of guidelines and knowledge in facing the pandemic, disruption in research over AMR and antibiotics stewardship eventuated in overuse and over‐prescription of antibiotics.^15^, ^20^

The current study showed an increase in resistance of Gram‐negative bacteria (Supporting Information: Figure 1), *P. aeruginosa*, *K. pneumonia*, *A. baumannii*, and *Citrobacter*, to most reported antibiotics by the pandemic presence, particularly in *P. aeruginosa* and *K. pneumonia*. A study in India on COVID‐19 patients reported an increase in resistance of *P. aeruginosa* to fourth generation of cephalosporins, including cefepime.^8^ The same research also notes a rise in ciprofloxacin and Gentamicin resistance in *K. pneumoniae*. Moreover, research conducted in Mexico found that both *K. pneumonia* and *P. aeruginosa* were becoming more resistant to cefepime, ciprofloxacin, and gentamicin.^18^

A recent study in Northeast Iran focusing on *E. coli, P. aeruginosa, K. pneumonia*, and *A. baumannii strains* showed a significant rise in resistance rate during 2020–2022. For instance, a 30% increase in *K. pneumonia* to cefotaxime is reported.^21^ Gram‐negative bacteria, specifically *K. pneumoniae*, are one of the main reasons for VAP in the intensive care units (ICU).^22^ On the other hand, the COVID‐19 pandemic had overwhelmed ICU admission due to respiratory failure. Prophylactic empirical prescription of antibiotics to control the threat of bacterial co‐infection led to an increase in AMR.^23^ Even though the risk of co‐infection was low, 70% of admitted COVID‐19 patients in Bangladesh received prophylactic antibiotics, according to a study. Long hospitalization and use of medical devices in the ICU, lack of effective surveillance, and immunocompromised patients due to corticosteroid prescription all contributed to an increase in hospital‐acquired Infection.^24^ Even more, the laboratory tests were in shortage ^23^ and nurse numbers were not in proportion to the patients,^25^ which may lead to the empirical use of antibiotics concluded in AMR increase.

Remarkably, during the pandemic, *A. baumannii* resistance to all mentioned antibiotics has been 100%, albeit it was not a frequent microorganism. Likewise, but not at the same level, *P. aeruginosa* and *Citrobacter* have had more than 50% resistance to the same antibiotics. It seems that these might be the most challenging post‐pandemic issues.

*E. coli* has different behavior in juxtaposition to other monitored Gram‐negative bacteria (Supporting Information: Figure 1), which might have emanated from the fact that the majority of *E. coli* specimens belong to urine for outpatients having a drastic fall the frequency during the pandemic. As observed by several studies in Indonesia 29 and Mexico, a rise in *E. coli*'s sensitivity to cotrimoxazole, ciprofloxacin, and ceftriaxone may have resulted from a decrease in outpatients.^18^ However, *E. coli* resistance to Gentamicin and cefepime, which are among the prescribed drugs during COVID‐19 in hospitals reported by some studies,^18^ has increased.

The increasing Gram‐positive bacteria' resistance to cephalexin may were caused by irrational overuse of it.^8^ The prevalence of Gram‐positive bacteria has decreased since COVID‐19's presence.^26^ Although the number of Gram‐positive samples on which antibiogram tests were done in the current study has not been enough to distinguish a significant trend, the increase in Gram‐positive pathogens' resistance specifically methicillin‐resistance *Staphylococcus aureus* and vancomycin‐resistance enterococcus must be a subject of a detailed study to be determined.

An increase in resistance to the cephalosporin third or fourth generation, such as ceftriaxone, cefotaxime, and cefepime (Supporting Information: Figures 1 and 2), could have been in terms of the indiscriminate use of them reported by two studies in China and Peru, 68% of COVID‐19 patients have had a history of azithromycin, and ceftriaxone administration before admission. Furthermore, 33% of them have had a self‐medication history.^27^, ^28^

The present study has some limitations, including a short period, and limited data; some antimicrobials were not evaluated in all classes, such as carbapenem, penicillin, methicillin, and azithromycin, which are some of the most important antibiotics to consider. Only the data of urine colony count were reported. The clinical status of patients, such as SARS‐COV‐2 infection was not reported. The information about prescribed antimicrobials during the COVID‐19 pandemic was not available. A strong point of the present study would be that all the data were collected from a COVID‐19 referral center. Furthermore, available data allowing us to compare prepandemic, and the pandemic period from the same center make the study more reliable about COVID‐19's effect on AMR. Focusing on some high‐priority, and critical bacteria turns this study into an outstanding one.

## 5 CONCLUSION

Although the accurate effect of COVID‐19 on AMR is not distinguishable yet, an increase in AMR was observed, particularly in Gram‐negative bacteria among which *P. aeruginosa* and *K. pneumonia* had a tremendous increase. It may have stemmed from excessive and inappropriate utilization of antibiotics. The lack of fully developed stewardship may have had an intensive effect on AMR increase. More strength surveillance may reduce irrational prescriptions of antibiotics. In addition, well‐developed guidelines to manage COVID‐19 patients, especially the mild ones, and appropriate diagnostic kits are recommended for detecting AMR early on to reduce empirical therapy. Moreover, the biomarkers to differentiate viral and bacterial infections avoid inappropriate antibiotic use. Further studies are required to determine bacterial co‐infection risk factors of COVID‐19 patients. The appliance of a hospital infection control team under a strict protocol can reduce the infection transmission, subsequently AMR. Public awareness to reduce self‐medication, and simultaneously, restriction of antibiotic accessibility over the counter may legally have an impressive effect on their consumption.

## AUTHOR CONTRIBUTIONS

**Rahim Raoofi**: Conceptualization; investigation; methodology; project administration; supervision; validation; writing—review & editing. **Negin Namavari**: Conceptualization; data curation; formal analysis; investigation; methodology; validation; visualization; writing—original draft. **Vahid Rahmanian**: Formal analysis; investigation; methodology; software; visualization; writing—original draft; writing—review & editing. **Mohammad Hadi Dousthaghi**: Conceptualization; data curation; formal analysis; investigation; software; validation; writing—original draft.

## CONFLICT OF INTEREST STATEMENT

The authors declare no conflict of interest.

## TRANSPARENCY STATEMENT

The lead author Negin Namavari affirms that this manuscript is an honest, accurate, and transparent account of the study being reported; that no important aspects of the study have been omitted; and that any discrepancies from the study as planned (and, if relevant, registered) have been explained.

## Supporting information

Supporting information.

## Data Availability

The authors acknowledge that data supporting the findings of this study are available in the article [and/or] its supplementary material.
