# Supplementary material for: Evaluation of antibiotics resistance in Southern Iran in light of COVID‐19 pandemic: A retrospective observational study
Source: Health Sci Rep. 2023 Mar 15;6(3):e1153. doi: 10.1002/hsr2.1153 (PMC10017310; doi:10.1002/hsr2.1153)
Supplement: Supplementary file 1 — Supporting information. [file HSR2-6-e1153-s001.docx]

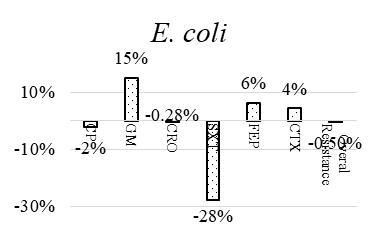

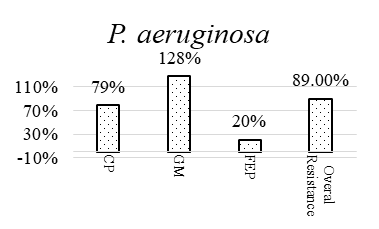

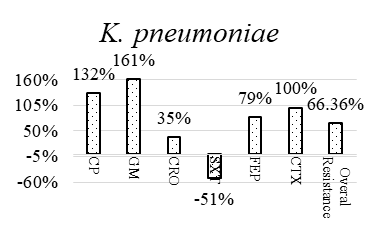

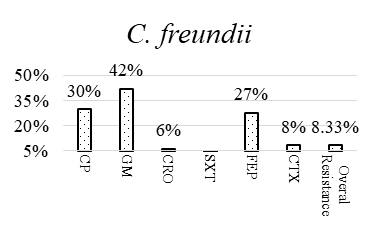

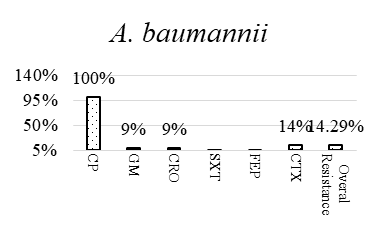

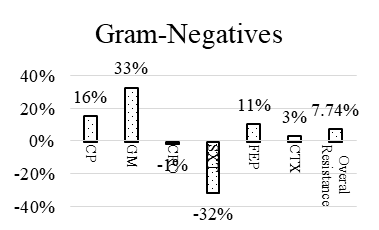


Suppliurementary Figure 1. The change rate of gram-negative bacteria to various antibiotics after the COVID-19 presence.

CP: Ciprofloxacin, GM: Gentamycin, CRO: Ceftriaxone, SXT: Cotrimoxazole, FEP: Cefepime, CTX: Cefotaxime


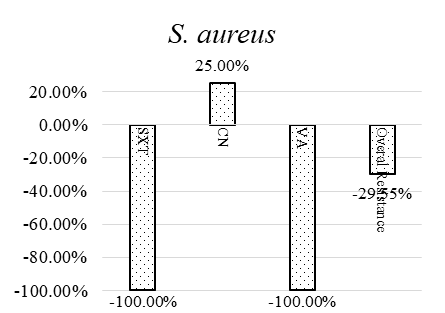

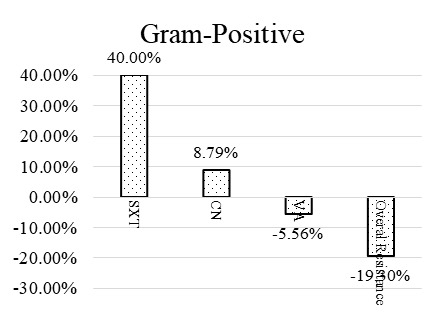


Suppliurementary Figure 2. The change rate of gram-positive microorganisms to various antibiotics after COVID-19 presence.

SXT: Cotrimoxazole, CN: Cephalexin, VA: Vancomycin,
